# A meta-analysis of working memory in individuals with autism spectrum disorders

**DOI:** 10.1371/journal.pone.0216198

**Published:** 2019-04-30

**Authors:** Abdullah Habib, Leanne Harris, Frank Pollick, Craig Melville

**Affiliations:** 1 College of Medical Veterinary and Life Sciences, Institute of Mental Health & Wellbeing, University of Glasgow, Glasgow, United Kingdom; 2 College of Science and Engineering, School of Psychology, University of Glasgow, Glasgow, United Kingdom; University of Macau, CHINA

## Abstract

**Background:**

Autism spectrum disorders (ASD) are lifelong neurodevelopmental disorders. It is not clear whether working memory (WM) deficits are commonly experienced by individuals with ASD.

**Aim:**

To determine whether individuals with ASD experience significant impairments in WM and whether there are specific domains of working memory that are impaired.

**Methods:**

We conducted a meta-analysis using four electronic databases EMBASE (OVID), MEDLINE (OVID), PsychINFO (EBSCOHOST), and Web of Science, to examine the literature to investigate whether people with ASD experience impairments related to WM. Meta-analyses were conducted separately for phonological and visuospatial domains of WM. Subgroup analyses investigated age and intelligence quotient as potential moderators.

**Results:**

A total of 29 papers containing 34 studies measuring phonological and visuospatial domains of WM met the inclusion criteria. WM scores were significantly lower for individuals with ASD compared to typically developed (TD) controls, in both the visuospatial domain when investigating accuracy (*d*: -0.73, 95% CI -1.04 to -0.42, p < 0.05) and error rates (*d*: 0.56, 95% CI 0.25 to 0.88, p<0.05), and the phonological domain when investigating accuracy (*d*:-0.67, 95% CI -1.10 to -0.24, p>0.05) and error rate (*d*: 1.45, 95% CI -0.07 to 2.96, p = 0.06). Age and IQ did not explain the differences in WM in ASD.

**Conclusions:**

The findings of this meta-analysis indicate that across the lifespan, individuals with ASD demonstrate large impairments in WM across both phonological and visuospatial WM domains when compared to healthy individuals.

## Introduction

Autism spectrum disorder (ASD) is a neurodevelopmental disorder characterized by communication difficulties, social impairment and fixated interests along with repetitive behaviours [1]. The symptoms of ASD are evident from young age; usually in children aged two or three, with a higher prevalence in boys than in girls [2]. Autism has become one of the most prevalent and common developmental disability, the Centers for Disease Control and Prevention [3] notes that the incidence of ASD has been increasing in the general population in recent years in the United States of America (USA), with the new estimate of 1 in 68 children having an ASD being roughly 30 percent higher than previous estimates reported in 2012, 1 in 88 children. In the United Kingdom (UK), according to Brugha et al. [4], 1.1% of the population in the UK had an ASD compared to 2009 when it was found 1% of the population studied had an ASD [5]. ASD has significant negative impact on the quality of life of the individual [6]. A meta-analysis [7] concluded that across the lifespan, quality of life is lower for people with ASD when compared to people without ASD. The impairments associated with ASD mean that many people with ASD remain dependent on others for support, such as parents, siblings, and other carers [8]. Thus, many parents of people with ASD are concerned about what to expect from the future and what will happen to their family members when they will not be able to take care of them anymore [9].

Impairments in cognitive abilities are not part of the classification of ASD. However, clinicians and researchers often make a distinction between low-functioning autism (LFA) with an intelligence quotient (IQ) below 65 or 70, and high-functioning autism (HFA) with an IQ above 65 or 70. Although neuropsychological impairments are not part of diagnostic criteria, many people with ASD experience significant cognitive impairments [10, 11, 12]. Executive function deficits are commonly experienced by individuals with ASD [11, 13, 14]. Executive function is an umbrella term for a set of cognitive processes that includes working memory (WM), inhibition, planning, impulse control, and shifting set as well as the initiation and monitoring of action [11].

WM plays an important role in human cognition and a central role in executive function [15]. The most commonly used cognitive model of WM is the revised WM model [16], which is based on the model developed by Baddeley and Hitch in 1974 [17]. The core of the model involves the central executive, concerned with information control and monitoring information processing (attention control center), an episodic buffer enables information integration from the sub-components of WM and long-term memory. Executive functions allow one to engage in purposeful and independent behaviours such as suppressing irrelevant information, shifting among multiple tasks, and revising and monitoring information held in long-term memory. The model also involves two storage systems- the phonological loop and the visuospatial sketchpad- supporting the central executive. The phonological loop provides temporary storage for phonological information while the visuospatial sketchpad allows temporary storage and manipulation of visual and spatial information. Other aspects of the model include the role of attention in WM and the concept of temporal duration when performing memory tasks. However, based on this revised WM model of Baddeley, WM is not only important but also essential for successfully navigating in the social world [18].

Gathercole and Baddeley describe WM as a short-term memory system that controls temporary processing and storage of information [19]. The importance and role of WM in everyday tasks is well established. WM plays a crucial role in supporting various complex high-level cognition activities such as language comprehension and long-term learning [19], reasoning [20], reading comprehension [21, 22], mental arithmetic [23], and problem solving [24]. As a temporary storage system under an individual’s attentional control, WM allows processing of complex cognitive information and plays central roles in social cognition, interpersonal interactions, and language comprehension. These roles make WM highly relevant in ASD because the disorder primarily concerns the cognitive domains involved in social impairments, communication problems, and repetitive activities [18]. Studies have shown that WM deficits in individuals with ASD are associated with learning disabilities [25], difficulties associated with behaviour regulation [26], cognitive flexibility, focusing and sustaining attention [27], abstract thinking [28], communication and socialising [29, 30], as well as restrictive and repetitive symptoms [31, 32]. Therefore, it is important to obtain a clearer and more accurate understanding of WM impairments in individuals with ASD as impairments in WM are associated with difficulties in everyday life and can have a negative impact on the quality of life.

Studies examining whether individuals with ASD experience significant WM impairments have produced inconsistent findings. Joseph, Steele, Meyer and Tager-Flusberg [33] examined verbal encoding and rehearsal strategies in the service of working memory in high-functioning children with autism and a comparison group. They found that while the two groups were equal in verbal rehearsal skills, the autism group performed significantly less in the verbal test, suggesting that children with ASD are deficient in the use of verbal mediation strategies to maintain and monitor goal-related information in working memory. Steele and colleagues tested high-functioning individuals with ASD on the Cambridge Neuropsychological Test Automated Battery (CANTAB) compared to a matched group of typically developing controls. Their findings suggest deficits in spatial working memory abilities in ASD and that these deficits are significant when tasks impose heavier demands on working memory [34]. Moreover, Morris et al, [35] investigated spatial working memory in ASD using the Executive Golf Task, where they found that The ASD group showed a substantial deficit on spatial working memory. Yerys and his team found a significant correlation between Consonant Trigrams Test (CTT) performance and everyday working memory, as CTT performance in children with ASD was significantly worse than in matched age and IQ controls [36]. So, several prominent studies have found that individuals with ASD experience WM impairments.

On the other hand, some studies have not reported significant WM impairments in individuals with ASD, Ozonoff et al, [37] investigated working memory in individuals with high-functioning autism, Tourette syndrome and a typically developing control group. No group differences were found across three tasks and five dependent measures of working memory, and it was concluded that working memory is not one of the executive functions that is seriously impaired in ASD. In another study, Russell and collegues [38] were unsuccessful in finding any significant group differences between children and adolescents with ASD as well as individuals with moderate learning difficulties and controls which were matched on mental age and on three measures of working memory capacity. Moreover, Faja and Dawson tested in 23 children with ASD without intellectual disability and 20 typically developing children matched on IQ and age on a backward digit span, and found that performance did not differ between groups [39]. Finally, the study by Griffith, Pennington, Wehner, & Rogers [40] which investigated spatial working memory in very young children with ASD and control groups matched on age, and verbal and nonverbal ability found no group differences across eight tasks which appeared to require working memory.

As described above, the findings from research on WM impairments in ASD has been inconsistent. One meta-analysis looking at WM in ASD has been published [41]. The authors reported a significant WM impairment and suggested that this impairment was not associated with age or IQ. They also demonstrated that spatial WM was more severely impaired than verbal WM and the component of cognitive processing (maintenance vs. maintenance plus manipulation) did not affect the severity of WM impairments. This initial meta-analysis flags up the relevance of research on WM and ASD. However, there were significant limitations in the methods used for the meta-analysis. A systematic literature search was not used to identify potential studies; only two search terms were used “Asperger+ working memory” and “autism + working memory”. A literature search that is not comprehensive can lead to relevant studies being missed and biased results from meta-analyses. In order to include studies that used error rate as the measure of WM, Wang et al [41] converted error rate into accuracy by assuming that error rate and accuracy have an opposite direction relationship. For example, if the error rate was 0.8 they converted it to an accuracy score of -0.8 (personal communication). This method is problematic as studies that have measured error rate and accuracy found that ASD participants’ accuracy scores did not differ from the control group however the ASD participants made more errors [33, 42]. For studies that had used more than one than one WM task, Wang et al [41] state that they calculated effect sizes for each WM task and then combined these into an unweighted average effect size. However, they excluded WM tasks from the average effect size calculation if participants with ASD did not demonstrate impairments on these tasks. For example, two studies measured reaction time and accuracy [43, 44] but the participants with ASD only had impairments on reaction time so the accuracy scores were excluded. Selection of studies based on the direction of the results creates bias and in this case will have inflated the overall effect size of the meta-analysis. These methodological weaknesses fall well short of guidance on the methods and reporting of systematic reviews and meta-analyses [45], and threaten the validity of the findings in the previous meta-analysis by Wang and colleges.

We previously explained the potential importance of WM in the daily functioning and quality of life of individuals with ASD. Our aim in this study is to determine whether individuals with ASD experience significant impairments in WM and whether there are specific domains of working memory that are impaired. We will also evaluate age and IQ as potential moderators of WM impairments in individuals with ASD.

To achieve these aims, in this systematic review and meta-analysis, we will address the limitations in the previous study [41] by adopting a more systematic and comprehensive search of the available literature, including more rigorous inclusion criteria that controls for matching participants on IQ and age (i.e., no significant difference between the groups) a more stringent selection process to identify relevant studies, avoiding bias by not using study results as the basis for inclusion, and analysing WM accuracy and error rates scores separately, as accuracy and error rate do not necessarily have an opposite relationship (i.e. if accuracy is high, error rate is low). Additionally, looking into only one of the outcomes would reduce the amount of studies included significantly as studies sometimes only report 1 of the outcomes. This was done in order to achieve a more accurate examination of the topic of WM impairments in individuals with ASD.

## Method

This study was conducted in adherence with the guidelines of the Preferred Reporting Items for Systematic Reviews and Meta-Analysis (PRISMA) [45].

### Literature search

We conducted a literature based search and manual cross referencing of English language empirical studies relating to both ASD and WM using four electronic databases EMBASE (OVID), MEDLINE (OVID), PsychINFO (EBSCOHOST), and Web of Science from 1986 to May 2017 (subsequent to a previous review by Wang et al. [41]). Search terms were combinations of the following ‘autis’, ‘asperg’, ‘pervasive development disorder’, ‘kanner’, ‘childhood schizophrenia’, ‘child development disorders’, ‘Rett’, ‘working memory’, ‘memory capacity’, ‘memory span’, ‘short-term memory’, ‘N-back’, ‘memory’, and ‘digit span’. The full search Medline search strategy is illustrated in the supporting information (S1 Appendix). The reference lists of retrieved studies were also examined to identify relevant papers.

### Inclusion criteria

Studies were eligible for this review if they met the following inclusion criteria:

- Published in peer-reviewed journals in English
- Included people with ASD
- Used ADOS [46], ADI- Revised [47], 3Di [48] or a clinician as a method to diagnosis ASD
- Matched the groups on age, gender and IQ or where there was no statistically significant difference between the groups
- Data reported clearly and sufficiently such as mean scores and standard deviation
- Compared ASD groups to TD groups
- Included a valid test of WM, the appropriateness of including tests as measures of WM was determined by referring to Lezak [49] or Baddeley, Wilson and Watts [50]

Research studies were not eligible for this review if they met the following exclusion criteria:

- Conference papers/abstracts
- Review papers
- Unpublished data, grey literature
- Non-English language papers.

### Selection of studies

The lead researcher (AH) performed the literature search and removed any duplicate studies. The titles and abstracts were screened independently by two authors (AH and CM) and disagreements about inclusion resolved at a consensus meeting. For records retained after screening, the full text was obtained, read in full and both researchers (AH and CM) independently completed an inclusion checklist. If there was any disagreement between the inclusion checklists for a paper final list decided decision about inclusion was made following a consensus discussion.

### Data extraction

The following data were extracted by the lead researcher to assess the methodology quality and data synthesis:

- Authors, year of publication
- Number of subjects
- Full scale IQ
- Age
- Gender
- Instruments used to assess WM
- WM scores (where there was multiple task being used, we chose the more challenging task, for example, they study by Williams et al. where multiple loads of the N-Back WM task were used (1-Back, 2-Back and 3-Back). The 3-back results were chosen as the 3-back is what is commonly used as a load when using N-back WM task which also happens to be the more challenging task.)
- The method of diagnosis was recorded for the ASD groups.

### Quality assessment

To check the quality of the studies, we used the Standard Quality Assessment Criteria for Evaluating Primary Research Papers tool for quantitative studies developed by Kmet, Lee, and Cook [51]. Each study was assessed against 14 criteria-oriented items. Criteria 5 (if interventional and random allocation was possible, was it described?), 6 (if interventional and blinding of investigators was possible, was it reported?) and 7 (if interventional and blinding of subjects was possible, was it reported?), were not considered during the quality assessment as they are applicable to studies assessing interventions. If the study met the criteria it was scored as 2; 1 if it partially met the criteria; and 0 if it did not meet the criteria. A total score for each study was calculated by adding the score across the criteria and dividing by the total possible score (22). The assessment was completed by two authors (AH and CM) for each study to improve reliability. There was complete agreement between the two reviewers.

### Data analysis

Meta-analyses were performed using Comprehensive Meta-analysis version 3.0 (Biostat, Englewood, NJ, USA). Effect sizes were calculated (using means, standard deviations and sample sizes) based on the pooled standardised mean difference (SMD), expressed as Cohen’s d [52] and 95% confidence interval (CI). Although studies measured the same outcome of WM, due to the different methodological tests to assess WM, it was necessary to standardise the results on a uniform scale (in order to combine results in the meta-analysis). The effect size was calculated as the difference in mean change between the ASD group and the TD/comparison group divided by the standard deviation pooled between the two groups. Effect sizes were interpreted as small (*d* = 0.20), moderate (*d* = 0.50) and large (*d* = 0.80).

WM was divided into subgroups via the following WM constructs, phonological and visuospatial, consistent with the gold-standard criterion recommendations [53]. Study results were pooled using an inverse variance weighted method of random effects analysis [54]. The significance and degree of heterogeneity were calculated using Cochrane’s Q statistic and I^2^. Cochrane’s Q statistic provides a measure of the variance between the effect sizes (with p < 0.05 illustrating evidence of heterogeneity) while I^2^ provides a measure of the amount of variance between the studies in terms of heterogeneity, and is described by Higgins et al. [55]. The degree of heterogeneity was measured by the *I*^2^ statistic, with *I*^2^ ≥ 50% indicating substantial heterogeneity. In accordance with the Cochrane handbook for reviews and to explore possible potential heterogeneity, subgroup analysis (post hoc) was conducted for variation in sample characteristics including moderator variables age and IQ considering all of our meta-analysis had 10 or fewer studies.

Publication bias was investigated using visual inspection of funnel plots of the SMD against the standard error of the SMD of the included studies and using the linear regression approach described by Egger et al. [56]. This method examines the association between effect size and standard error for each study and takes into account the sample size and effect size.

## Results

**Fig 1:**
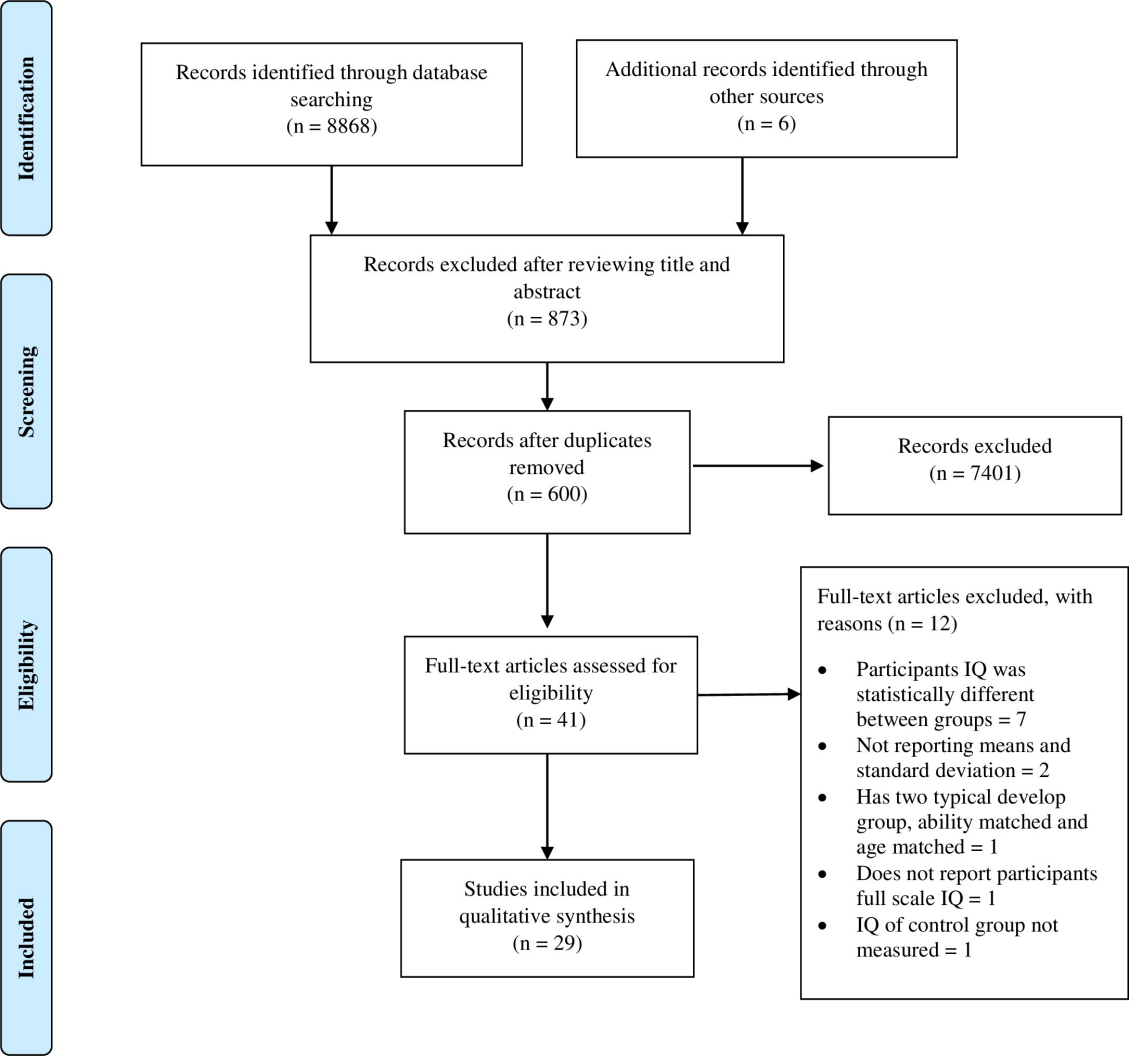
Flow diagram of study selection process in accordance with the PRISMA statement.

Of a total of 8868 studies, 273 duplicate studies were removed, 7995 articles were excluded on reviewing the title and abstract. For the remaining 600 full text articles, those that were conference papers, review articles or not in English, were excluded. We identified a total of 29 papers that evaluated WM performance for individuals with ASD; 16 investigated accuracy as a measure of participants working memory performance, while 13 investigated participant error rates. Five studies were excluded for not reporting the statistics efficiently, such as the means and standard deviations of each group, eight studies were excluded for not matching participants on IQ or there was a significant difference between the two groups, one study was excluded for not measuring full scale IQ, one study was excluded for not having a matched age and IQ control group, one study was excluded for not having a control group, finally, one study was excluded for not measuring the IQ of the control group. Studies where we were not able to contact the authors and/or access their data were excluded from this review. The articles were obtained from 11 different journals and were published between 2001 and 2015. A total of 29 papers containing 34 studies were retained for inclusion in the review and data synthesis. Based on the WM model by Baddeley [17] results were categorised based on which aspect of WM was tested, phonological or visuospatial. Fig 1 shows the study selection process.

### Phonological working memory

#### Accuracy in phonological working memory

Out of the 34 studies, nine studies were identified testing accuracy in phonological WM. A summary of the study characteristics of the nine studies is presented in Table 1. The studies were published between 2001 and 2013 in nine different journals. A total of 447 participants were recruited (226 ASD, 221 TD) across the nine studies, with a mean total ASD sample size of 25.1 and TD sample size of 24.5. Participants’ ages ranged from 11 to 31 years with the mean age of ASD participants of 20.7 years and TD participants ages ranged from 11 to 38 years with the mean age of 21.2 years. All nine studies compared ASD participants with TD participants with all participants’ IQ scores being above 70.

**Table 1:** Main characteristics of accuracy in phonological WM studies included in the meta-analysis.

Author	Cohen’s *d*	Variance	ASD N	ASD mean age	TD N	TD mean age	ASD WM scores	TD WM scores	ASD FSIQ	TD FSIQ	Diagnosis	WM assessment
Gonzalez-Gadea et al., 2013	0.13	0.09	23	33.00	21	28.29	5.61 (1.31)	5.43 (1.47)	37.43	37.14	DC	BDC
Gracia-Villamisar et al., 2002	-2.98	0.26	16	23.50	16	21.19	48.13 (16.77)	86.88 (7.58)	42.75	43.69	DC	DR
Ham et al., 2011	-0.40	0.10	19	12.10	23	12.00	98.60 (20.20)	107.00 (21.60)	106.00	111.40	ADOS	DR
Maister et al., 2011	-0.61	0.14	15	11.80	15	11.20	30.30 (9.00)	36.10 (10.10)	39.70	40.00	ADI-R	PWS
Minshew and Goldstein, 2001	-0.42	0.05	52	22.33	40	21.55	1.72 (1.44)	2.36(1.62)	92.88	96.53	ADI and ADOS	S-TWM
Poirier et al., 2011	-0.96	0.14	16	31.60	16	34.80	0.64 (0.20)	0.81 (0.15)	100.30	102.40	ADOS	F-BDC
Schuh et al., 2012	-1.27	0.13	18	12.00	18	13.00	16.00 (2.00)	18.00 (1.00)	105.00	104.00	ADI and ADOS	LNS
Williams et al., 2006	-0.22	0.05	38	11.68	38	12.16	8.61 (3.33)	9.26 (2.61)	103.82	107.18	ADI and ADOS	WRAML
Williams et al., 2005 b	-0.20	0.06	29	28.73	34	26.53	10.86 (3.07)	11.38 (2.24)	105.86	109.65	ADI and ADOS	WRAML

Note: ASD: Autism spectrum disorder; TD: Typically developing; FSIQ: Full scale intelligence quotient; N: Number; WM: Working memory;ADI: Autism Diagnostic Interview; ADOS: Autism Diagnostic Observation Schedule; DC: Diagnosed by a clinician; S-TWM: Three-word short-term memory task; WRAML: WMS–III, the Wide Range Assessment of Memory and Learning; DR: Digit recall; BDC: Backward digit recall; F-BDC: forward and backward digit recall; PWS: Phonological word-span task; LNS: Letter-Number Sequencing subtest from the Wechsler Intelligence Scale for Children, 4th Edition.

**Fig 2:**
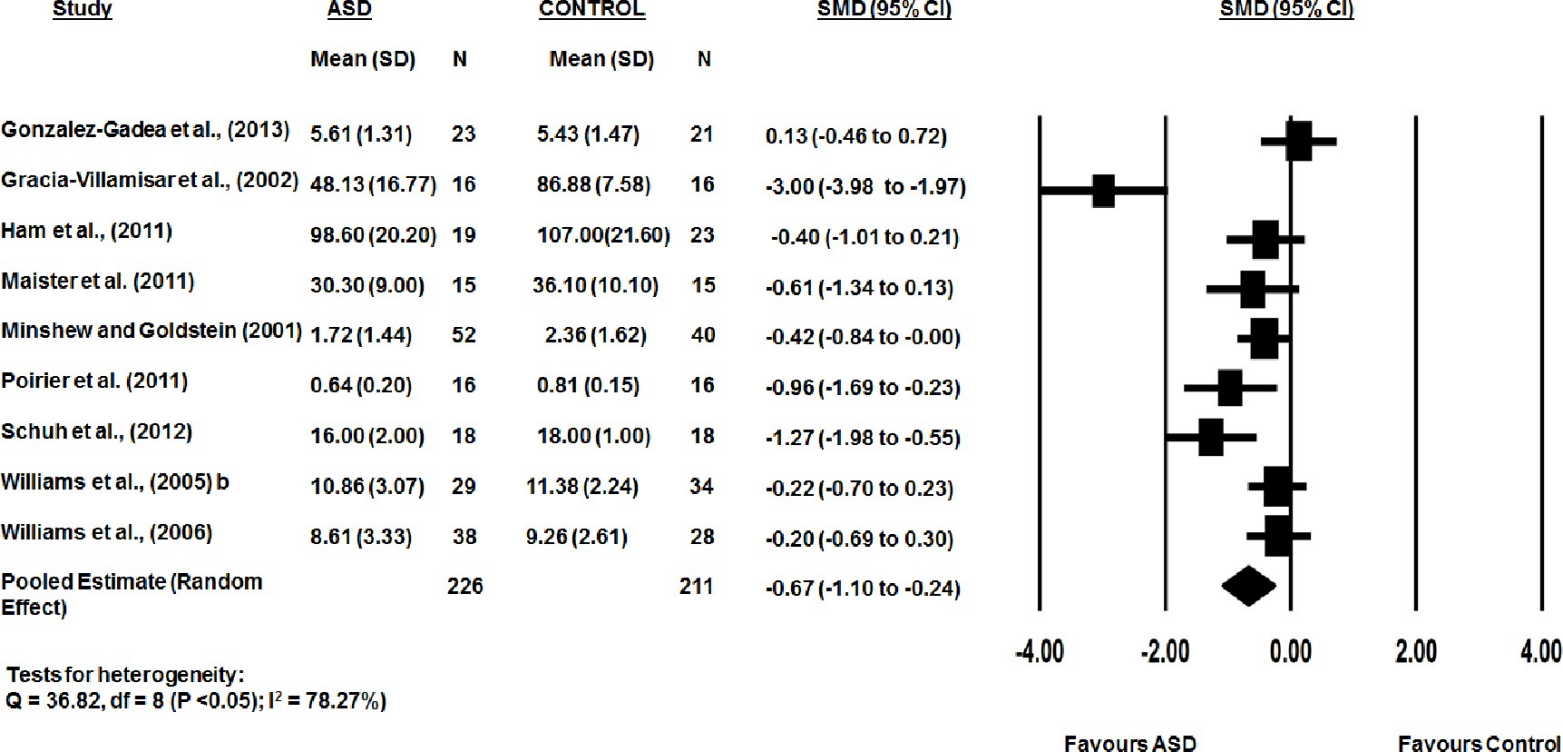
Accuracy in phonological WM between ASD and typically developing controls.

The combined WM scores from the nine studies were significantly lower in the ASD group than the typical developed group (*d*:-0.67, 95% CI -1.10 to -0.24, p<0.05). There was substantial heterogeneity between studies (Q-statistic = 36.82, df = 8 (p < 0.05); I^2^ = 78.27%). As only nine studies were identified as testing accuracy in phonological WM, publication bias was not assessed. This was due to the limited number of studies to provide adequate power of reliability of tests to detect for presence of publication bias [57]. Representative forest plots from the phonological WM meta-analyses are shown in Fig 2.

#### Error in phonological working memory

Out of the 13 studies, two studies were identified testing error in phonological WM. A summary of the study characteristics of the two studies is presented in Table 2. A total of 80 participants were recruited (45 ASD, 35 TD) across the two studies, with a mean total ASD sample size of 22.5 and TD sample size of 17.5. Participants’ ages ranged from 10 to 31 years with the mean age of ASD participants of 20.9 years and TD participants ages ranged from 11 to 32 years with the means age of 21.5 years. Both compared ASD participants with TD participants with all participants’ IQ scores being above 70.

**Table 2:** Main characteristics of error rate in phonological working memory studies included in the meta-analysis.

Author	Cohen’s *d*	Variance	ASD N	ASD mean age	TD N	TD mean age	ASD WM scores	TD WM scores	ASD FSIQ	TD FSIQ	Diagnosis	WM assessment
Williams et al., 2014	0.68	0.12	17	31.06	17	31.92	0.21 (0.32)	0.05 (0.09)	114.10	117.70	ADOS and DC	PM Task
Yerys et al., 2011	2.22	0.15	28	10.89	18	11.07	65.36 (11.50)	43.11 (7.00)	113.90	118.90	DC, ADI and ADOS	CTT

Note: ASD: Autism spectrum disorder; TD: Typically developing; FSIQ: Full scale intelligence quotient; N: Number; WM: Working memory; ADI: Autism Diagnostic Interview; ADOS: Autism Diagnostic Observation Schedule; DC: Diagnosed by a clinician; PM Task: Prospective memory task; CTT: Consonant trigrams test.

**Fig 3:**
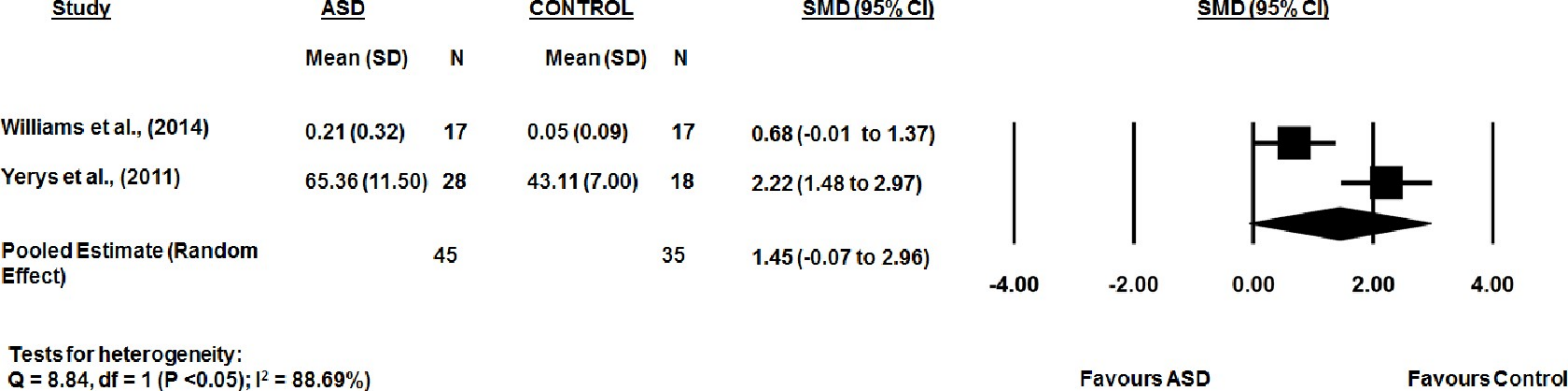
Error rate for ASD and typical developed controls groups in phonological WM.

The WM error rates scores from the two studies were significantly lower in the TD group than the ASD group (d: 1.45, 95% CI -0.07 to 2.96, p = 0.06). There was substantial heterogeneity between studies (Q-statistic = 8.84, df = 1, (p<0.05); I2 = 88.69%). Publication bias was also not assessed for studies testing error in phonological WM. Representative forest plots from the phonological WM meta-analyses are shown in Fig 3.

#### Subgroup analysis of phonological working memory

The results above show that there was a significant impairment in both accuracy and error rate in phonological WM in people with ASD. However, to examine whether this effect was consistent across lifespan and to explore the variation in effect sizes post-hoc subgroup analysis was performed using age and IQ as moderators (Table 3). Age was dichotomised into children (< 18 years) and adults (≥ 18 years). There were four studies that investigated accuracy in phonological memory in children and five phonological memory in adults. There were no between group differences in age (Q = 0.25; p = 0.62) for accuracy in phonological WM (adults: *d* -0.79, 95% CI, -1.53 to -0.04 vs child: *d* -0.57, 95% CI, -1.01 to -0.13). Mean IQ of study participants was dichotomised (average 90–109; high average 110–119). The accuracy phonological WM scores for all participants was reported as all having a mean average IQ and therefore not divided into subgroups. Moreover, as only two studies measured error in phonological WM, subgroup analysis was not conducted.

**Table 3:** Subgroup analysis phonological WM.

Study or Subgroup					Heterogeneity			
	K	SMD	95% CI	p-value	Q_model_	P-value (Q_model_)	I^2^	Q_between_ (p-value)
***Phonological Accuracy***								
Adult	5	-0.79	-1.53 to -0.04	0.038	30.74	<0.001	86.99	0.25 (0.62)
Children	4	-0.57	-1.01 to -013	0.0111	6.08	0.11	50.66	

Note: K = number of studies; WM: Working memory; SMD = standardised mean difference; CI = confidence interval; Q_model_ = heterogeneity statistic for the model; I^2^ = index of heterogeneity beyond within-study sampling error; Q_between_ = between-groups heterogeneity statistic

### Visuospatial working memory

#### Accuracy in visuospatial working memory

Twelve studies tested accuracy in visuospatial WM. A summary of the study characteristics of the twelve studies is presented in Table 4. The studies were published between 2005 and 2015 and included 12 different journals. A total of 656 participants were recruited (305 ASD, 351 TD) across the 12 studies, with a mean total ASD sample size of 23.5, and TD sample size of 27. Participants’ ages ranged from 11 to 63 years for ASD with a mean of 25.4 years, and TD age ranged from 10 to 63 with a mean age of 25.4 years. All twelve studies compared ASD participants with TD participants with all participants IQ scores being within typical range of 70 or greater.

**Table 4:** Main characteristics of accuracy in visuospatial WM studies included in the meta-analysis.

Author	Cohen’s *d*	Variance	ASD N	ASD mean age	TD N	TD mean Age	ASD WM scores	TD WM scores	ASD FSIQ	TD FSIQ	Diagnosis	WM assess-ment
Brenner et al. (2015)	-0.45	0.08	27	12.68	25	13.41	9.54 (2.80)	10.82 (2.91)	101.31	106.96	ADI and ADOS	TRT
Crane et al. (2013)	-.067	0.08	28	41.57	28	40.53	10.16 (3.13)	11.89 (1.92)	117.18	115.11	DC	WMS-III
Cui et al. (2010)	-0.93	0.13	12	7.46	29	7.37	8.00 (1.76)	9.28 (1.19)	100.03	108.31	DC	BR and VPT
Geurts and Vissers (2012)	-0.88	0.10	23	63.60	23	63.70	6.60 (1.70)	8.10 (1.70)	109.50	109.80	DC	WMS-III
Jiang et al. (2014)	-1.96	0.14	21	11.00	21	10.90	1.51 (0.30)	2.01 (0.20)	110.50	111.90	ADI and ADOS	SWMT
Maister et al. (2011)	-0.38	0.14	15	11.80	15	11.20	12.30 (2.50)	13.10 (1.60)	39.70	40.00	ADI and DC	MST
Nakahachi et al., 2006	-0.51	0.10	16	28.00	28	28.30	31.90 (12.30)	37.40 (9.70)	101.00	103.00	DC	ATMT
Schuh et al. (2012)	-1.00	0.13	18	12.00	18	13.00	9.00 (2.00)	11.00 (2.00)	105.00	104.00	ADI and ADOS	FW
Williams et al. (2005) a1	0.13	0.07	31	26.58	25	26.76	570.03 (128.91)	554.08 (121.34)	108.65	109.76		N-Back
Williams et al. (2005) a2	-0.32	0.07	24	11.75	44	12.39	576.79 (127.26)	623.14 (150.98)	109.67	109.95	ADI and ADOS	N-Back
Williams et al. (2005) b	-1.58	0.08	29	28.73	34	26.53	7.28 (3.02)	11.85 (2.79)	105.86	109.65	ADI and ADOS	WMS-III
Williams et al. (2006)	-0.47	0.05	38	11.68	38	12.16	8.63 (2.83)	9.92 (2.66)	103.82	107.18	ADI and ADOS	WMS-III

Note: ASD: Autism spectrum disorder; TD: Typically developing; FSIQ: Full scale intelligence quotient; N: Number; WM: Working memory; ADI: Autism Diagnostic Interview; ADOS: Autism Diagnostic Observation Schedule; DC: Diagnosed by a clinician; BR: Block recall; ATMT: Advanced Trail Making test; VVT: variant-visual-pattern test; MTS: A visuo-spatial delayed match-to-sample task; FW: Finger Windows subtest from the Wide Range Assessment of Memory and Learning; WMS-III: Wechsler Memory Scale; SWMT: Spatial working memory task; TRT: The time reproduction task.

**Fig 4:**
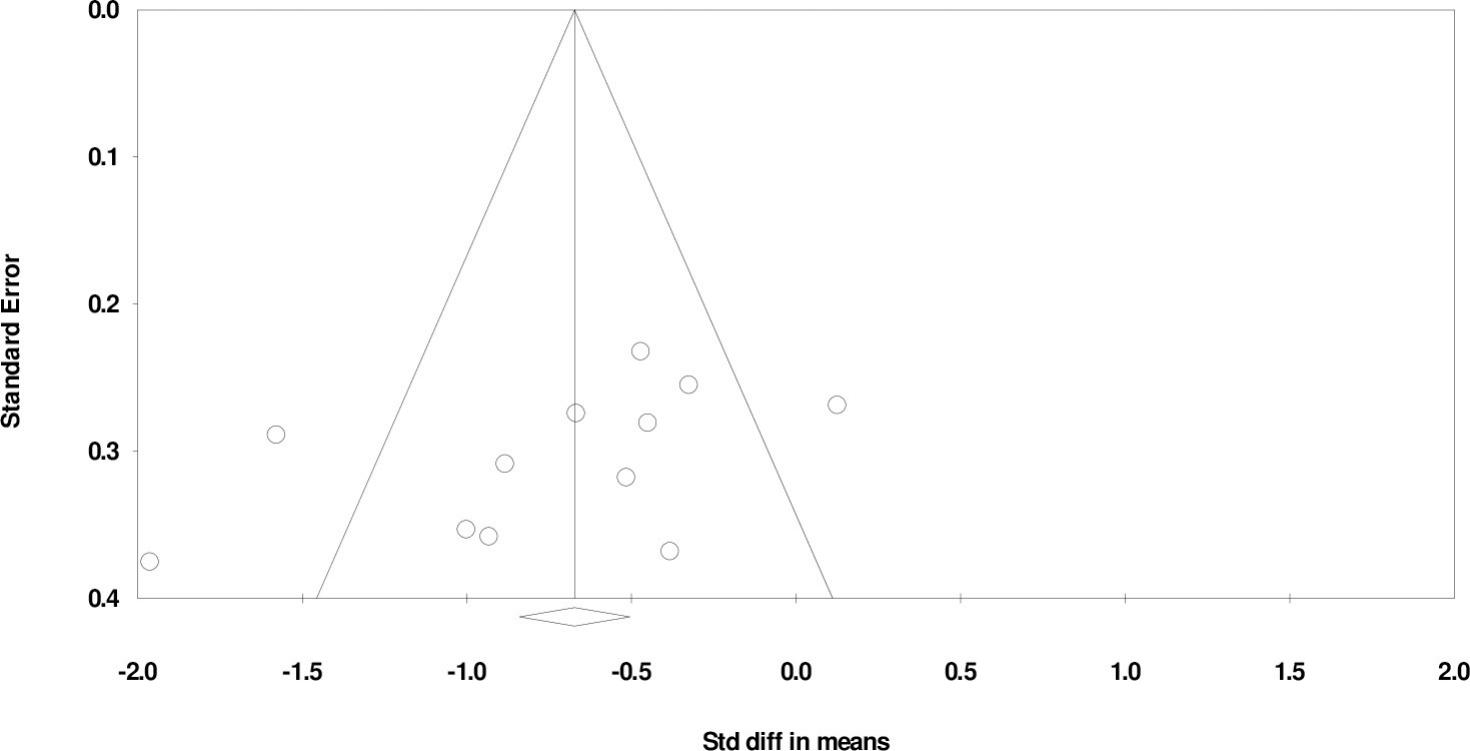
Funnel plot for accuracy in visuospatial working memory, Egger’s linear regression P = 0.09. SMD effect size plotted against standard error. The circles represent the studies in the analysis. The vertical line represents the population effect estimate and the diagonal lines represent the 95% confidence intervals.

**Fig 5:**
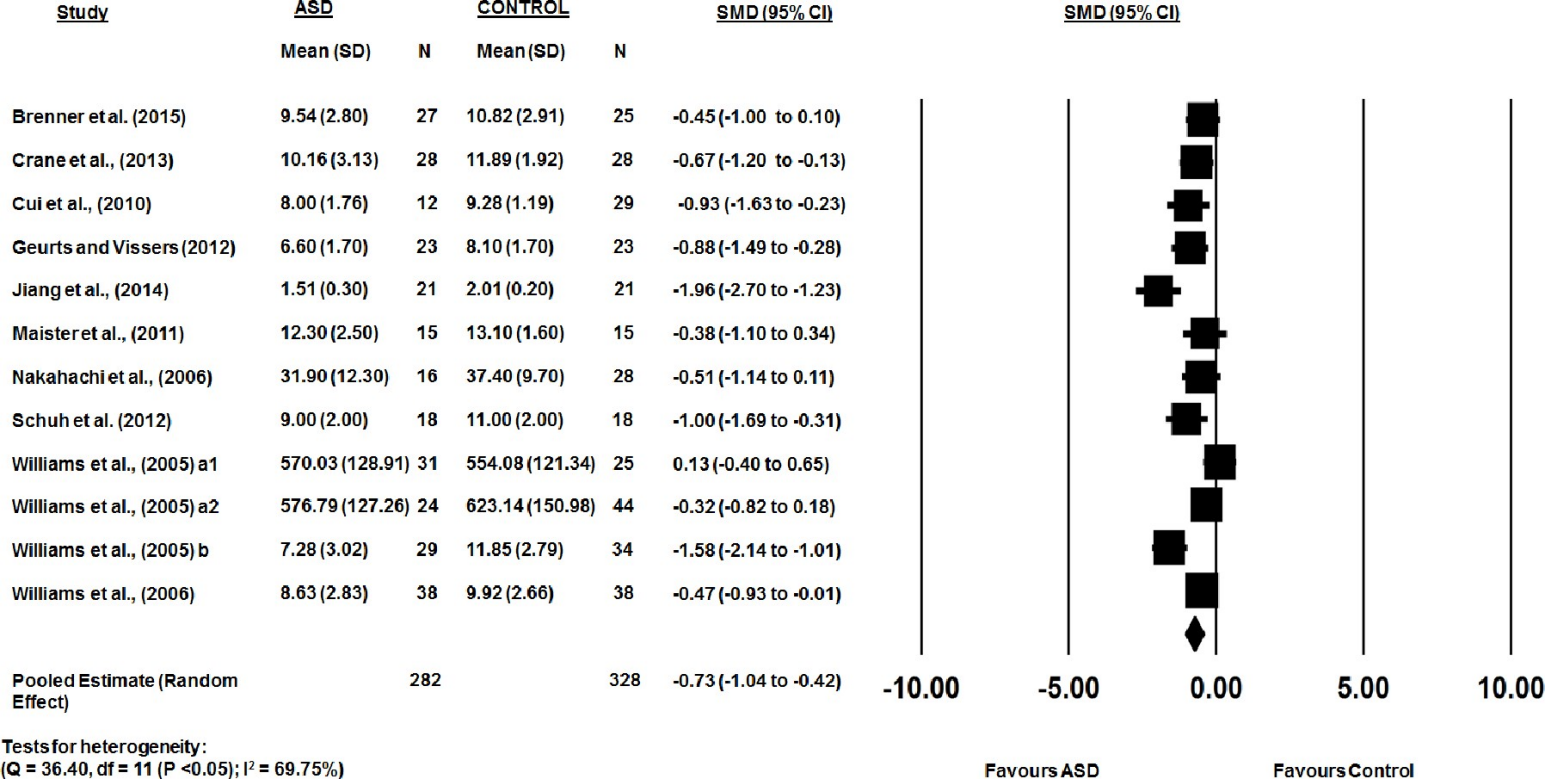
Accuracy in visuospatial WM between ASD and typically developing controls.

The combined WM scores from the 12 studies were significantly lower in the ASD group than the TD group (*d*: -0.73, 95% CI -1.04 to -0.42, p < 0.05). There was a substantial heterogeneity between studies (Q-statistic = 36.40, df = 11 (P <0.05); I^2^ = 69.75%) with a statistically insignificant publication bias (Egger’s linear regression P = 0.09; Fig 4). Representative forest plots from the phonological WM meta-analyses are shown in Fig 5. Comparison of Figs 2 and 5 appear to suggest that there is a greater impairment in the visuospatial domain when measuring accuracy.

#### Error in visuospatial working memory

Eleven studies tested error in visuospatial WM. A summary of the study characteristics of the eleven studies is presented in Table 5. The studies were published between 2005 and 2014 and published between nine different journals. A total of 691 participants were recruited (342 ASD, 349 TD) across the eleven studies, with a mean total ASD sample size of 31.1, and TD sample size of 31.7. Participants’ ages ranged from 8 to 28 years, with the ASD mean of 13.8 (range 8–24), and TD mean age of 14.4 (range 8–28). All twelve studies compared ASD participants with TD participants with all participants IQ scores being within typical range of 70 or greater.

**Table 5:** Main characteristics of error rate in visuospatial WM studies included in the meta-analysis.

Author	Cohen’s *d*	Variance	ASD N	ASD mean age	TD N	TD mean age	ASD WM scores	TD WM scores	ASD FSIQ	TD FSIQ	Diagnosis	WM assessment
de Vries and Geurts 2014	0.44	0.03	79	10.70	71	10.30	9.60 (6.10)	7.30 (3.90)	109.30	107.70	ADI and DC	N-Back
Happe et al., 2006	0.59	0.07	32	10.90	32	11.20	46.90 (8.80)	42.30 (6.60)	99.70	106.80	DC	CANTAB
Jospeh et al., 2005	0.70	0.09	24	8.11	24	8.11	5.60 (2.70)	3.90 (2.10)	96.00	92.00	ADOS, ADI and DC	SOPT
Kaufmann et al., 2013	0.57	0.21	10	14.70	10	13.80	24.60 (19.50)	14.60 (15.60)	102.30	109.50	ADOS and ADI	CANTAB
Koshino et al., 2008	-1.01	0.21	11	24.50	11	28.70	12.50 (2.90)	15.90 (3.80)	104.50	108.60	ADOS and ADI	N-Back Faces
Landa and Goldberg 2005	1.02	0.12	19	11.01	19	11.00	52.70 (17.90)	35.80 (15.30)	109.70	113.40	ADOS and ADI	CANTAB
Sachse et al., 2013	0.99	0.08	30	19.20	28	19.90	33.30 (22.20)	15.60 (11.80)	105.30	109.30	ADOS, ADI and DC	CANTAB
Sinzig et al., 2008	-0.54	0.10	20	14.30	20	13.10	-0.62 (1.31)	0.01 (1.00)	112.00	113.00	DC	CANTAB
Solomon et al., 2009	0.98	0.10	22	182.00	23	191.00	0.26 (0.20)	0.11 (0.09)	107.00	113.00	ADOS and DC	Pop task
Steele et al., 2007	1.02	0.08	29	14.83	29	16.93	0.17 (0.11)	0.08 (0.06)	107.80	110.80	ADOS and ADI	CANTAB
Verte et al., 2006	0.90	0.03	66	8.70	82	9.20	21.1 (7.70)	14.90 (6.20)	101.50	112.20	ADI and DC	SOPT

Note: ASD: Autism spectrum disorder; TD: Typically developing; FSIQ: Full scale intelligence quotient; N: Number; WM: Working memory; ADI: Autism Diagnostic Interview; ADOS: Autism Diagnostic Observation Schedule; DC: Diagnosed by a clinician; CANTAB: Cambridge Neuropsychological Test Automated Battery; POP: Preparing to Overcome Prepotency; SOPT: Self-ordered pointing task.

**Fig 6:**
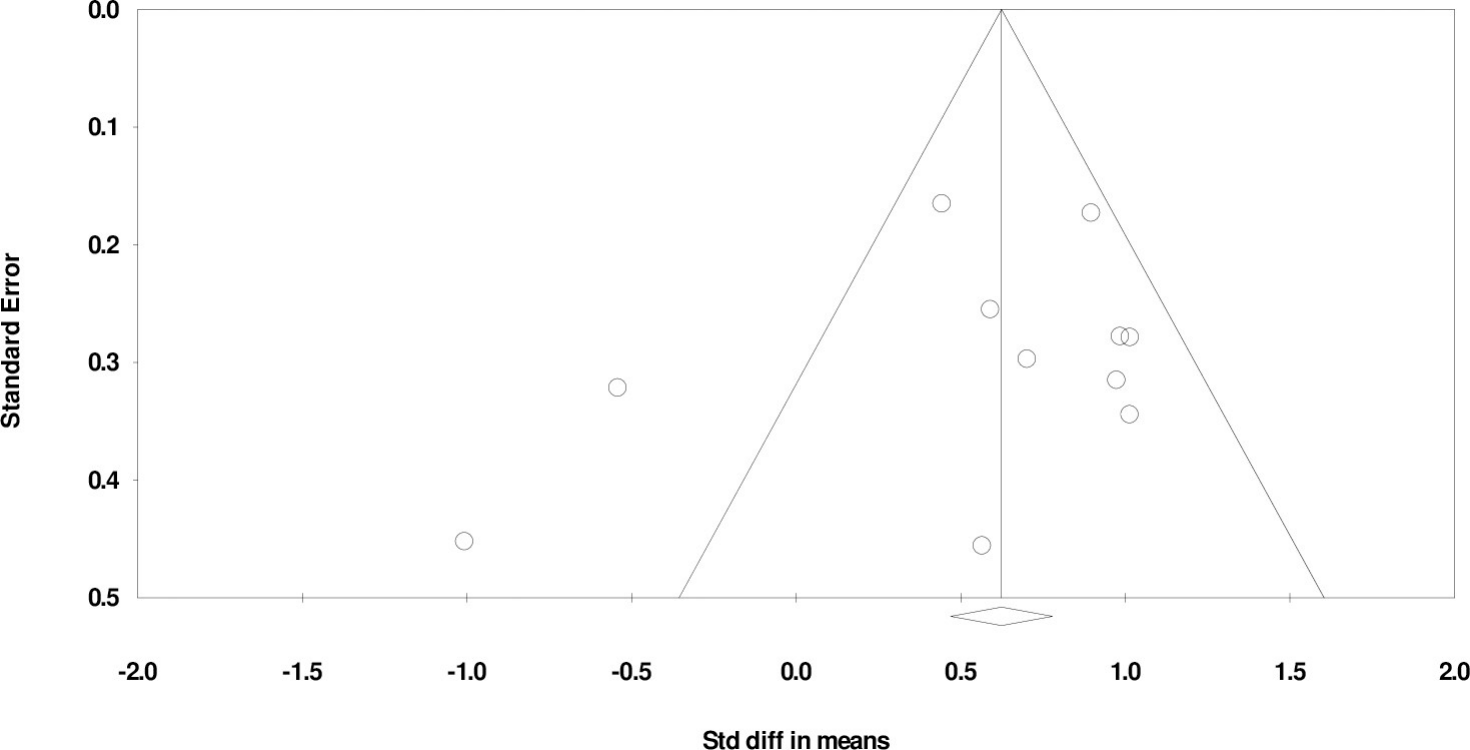
Funnel plot for error rate in visuospatial working memory, Egger’s linear regression P = 0.4. SMD effect size plotted against standard error. The circles represent the studies in the analysis. The vertical line represents the population effect estimate and the diagonal lines represent the 95% confidence intervals.

**Fig 7:**
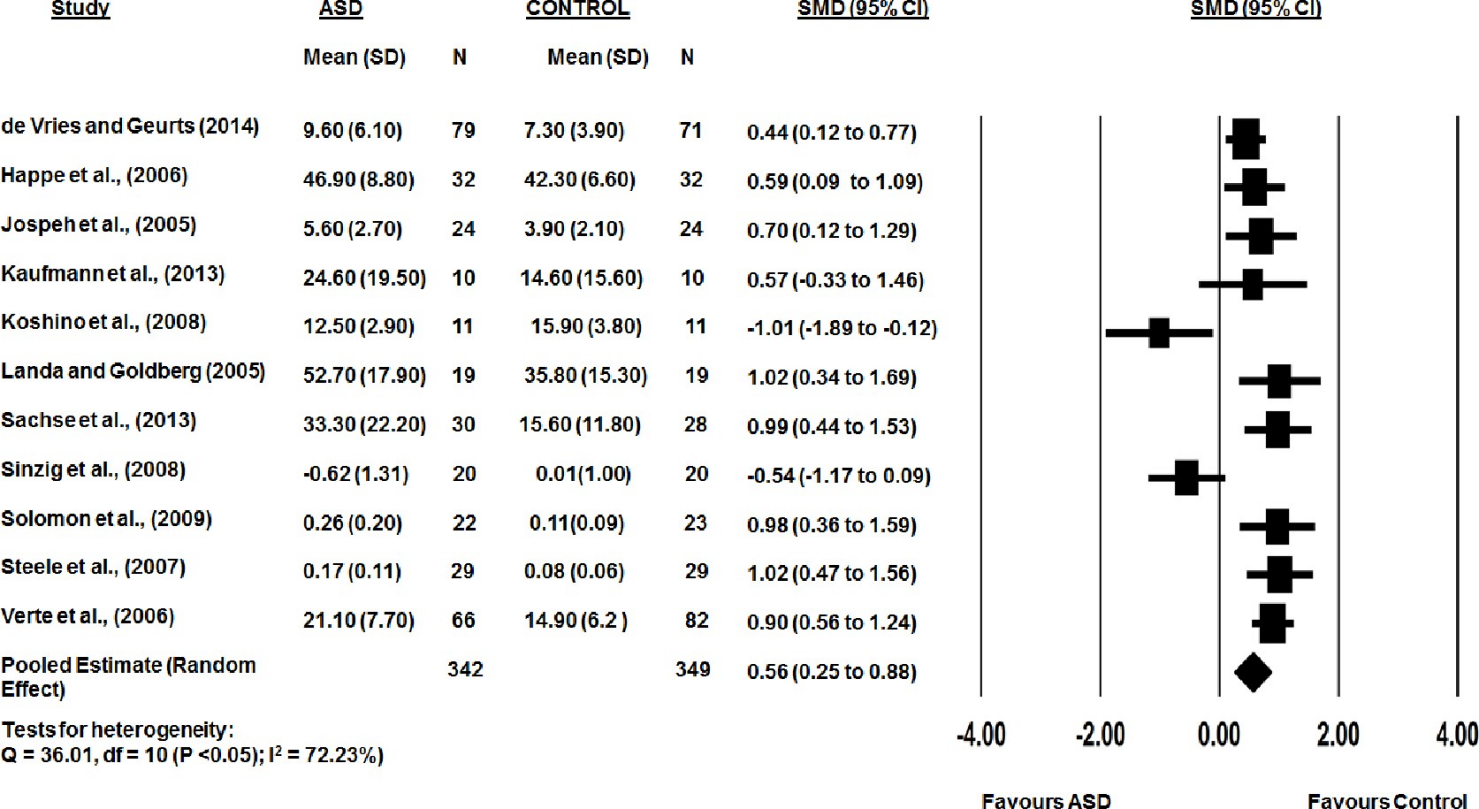
Error rate in visuospatial WM between ASD and typically developing controls.

The combined WM error rate scores from the eleven studies were significant lower in the TD group than the ASD group (*d*: 0.56, 95% CI 0.25 to 0.88, p<0.05). There was a substantial heterogeneity between studies (Q-statistic = 36.01, df = 10 (P < 0.05); I^2^ = 72.23%) with statistically insignificant publication bias (Egger’s linear regression P = 0.4; Fig 6). Representative forest plots from the visuospatial WM meta-analyses are shown in Fig 7.

#### Subgroup analysis of visuospatial working memory

Eight studies investigated visuospatial accuracy in children, four in adults and nine studies measured visuospatial error rate in children, two in adults, presented in Table 6. There, were no between group differences in age (Q = 1.67; p = 0.20) for accuracy in visuospatial WM (adults: *d* -0.47, 95% CI, -0.91 to -0.03) vs child: *d* -0.86, 95% CI, -1.27 to -0.46) or in age (Q = 0.38; p = 0.54) for error in visuospatial WM (adults: *d* 0.02, 95% CI, -0.93 to 1.97 vs child: *d* 0.64, 95% CI, 0.35 to 0.92).

**Table 6:** Subgroup analysis visuospatial WM.

Study or Subgroup					Heterogeneity			
	K	SMD	95% CI	p-value	Q_model_	P-value (Q_model_)	I^2^	Q_between_ (p-value)
***Visuospatial Accuracy***								
Adult	4	-0.47	-0.91 to -0.03	0.037	7.22	0.07	58.44	1.63 (0.220)
Children	8	-0.86	-1.27 to -0.46	<0.001	25.58	0.001	72.63	
Average IQ	10	-0.62	-0.93 to -0.32	<0.001	23.88	0.004	62.31	
High Average IQ	2	-1.29	-2.56 to -0.02	0.046	7.75	0.005	87.09	1.00 (0.32)
***Visuospatial Error rate***								
Adult	2	0.02	-1.93 to 1.97	0.982	14.06	<0.001	92.89	0.38 (0.54)
Children	9	0.64	0.35 to 0.92	<0.001	21.27	0.006	62.40	
Average IQ	8	0.61	0.30 to 0.92	<0.001	20.26	0.005	65.44	
High Average IQ	3	0.48	-0.53 to 1.49	0.351	14.91	0.001	86.59	0.05 (0.81)

Note: K = number of studies; WM: Working memory; SMD = standardised mean difference; CI = confidence interval; Q_model_ = heterogeneity statistic for the model; I^2^ = index of heterogeneity beyond within-study sampling error; Q_between_ = between-groups heterogeneity statistic

Two studies included participants categorised as having high average IQ (*d*: -1.29; 95% CI -2.56 to -0.02) and ten studies included participants with average IQ (d: -0.62; 95% CI -0.93 to -0.32) in accuracy visuospatial WM. There was no between group difference in accuracy in visuospatial WM (Q = 1.00; p = 0.32). Three studies involved participants categorised as having high average IQ (d: 0.48; 95% CI -0.53 to 1.49) and eight studies with participants with average IQ (d: 0.61; 95% CI 0.30 to 0.92) in error visuospatial WM. There was no significant between group difference for error in visuospatial WM (Q = 0.05.; p = 0.81).

#### Quality assessment

Assessment scores were converted to a percentage score, scores ranged from 81 to 100%. Nineteen studies were assessed as very good quality and were scored 22/22 = 100% and 21/22 = 95%. Ten studies were assessed as good quality and were scored 20/22 = 91%, 19/22 = 86%, and 18/22 = 81%. Results are presented in Table 7 along with scores from the quality assessment checklist. All papers were considered of sufficient quality.

**Table 7:** Quality assessment.

Study	Question / objective sufficiently described?	Study design evident and appropriate?	Method of subject/ comparison group selection or source of information/ input variables described and appropriate?	Subject (and comparison group, if applicable) characteristics sufficiently described?	Outcome and (if applicable) exposure measure(s) well defined and robust to measurement / misclassification bias? Means of assessment reported?	Sample size appropriate?	Analytic methods described/ justified and appropriate?	Some estimate of variance is reported for the main results?	Controlled for confounding?	Results reported in sufficient detail?	Conclusions supported by the results?	Total score	Percentage
Brenner et al. (2015)	2	2	2	2	2	2	2	2	2	2	2	22	100%
Crane et al. (2013)	2	2	2	2	2	2	2	2	2	2	2	22	100%
Cui et al. (2010)	2	2	2	2	2	0	2	2	2	2	2	20	91%
de Vries and Geurts (2014)	2	2	2	2	2	2	2	2	2	2	2	22	100%
Geurts and Vissers (2012)	0	2	2	2	2	2	2	2	2	2	2	20	91%
Gonzalez-Gadea et al., (2013)	2	2	2	2	2	2	2	2	2	2	2	22	100%
Gracia-Villamisar (2002)	2	2	2	2	2	2	2	2	2	2	2	22	100%
Ham et al., (2011)	0	2	2	2	2	2	2	2	2	2	2	20	91%
Happe et al., (2006)	2	2	2	2	2	1	2	2	2	2	2	21	95%
Jiang et al. (2014)	2	2	2	2	2	2	2	2	2	2	2	22	100%
Jospeh et al., (2005)	1	2	2	2	2	2	2	2	2	2	2	21	95%
Kaufmann et al., (2013)	2	2	2	2	2	0	2	2	2	2	1	19	86%
Koshino et al., (2008)	1	2	2	2	2	0	2	2	2	2	2	19	86%
Landa and Goldberg (2005)	2	2	2	2	2	2	2	2	2	2	2	22	100%
Maister et al. (2011)	2	2	2	2	2	2	2	2	2	2	2	22	100%
Minshew and Goldstein (2001)	2	2	2	2	2	2	2	2	2	2	2	22	100%
Nakahachi et al., (2006)	2	2	2	2	2	2	2	2	2	2	2	22	100%
Poirier et al. (2011)	0	2	2	2	2	2	2	2	2	2	2	20	91%
Sachse et al., (2013)	2	2	2	2	2	2	2	2	2	2	2	22	100%
Schuh et al. (2012)	2	2	2	2	2	2	2	2	2	2	2	22	100%
Sinzig et al., (2008)	2	2	2	2	2	2	2	2	2	2	2	22	100%
Solomon et al., (2009)	2	2	2	2	2	2	2	2	2	2	2	22	100%
Steele et al., (2007)	0	2	2	2	2	2	2	2	2	2	2	20	91%
Verte et al., (2006)	1	2	2	2	2	2	2	2	0	2	2	19	86%
Williams et al (2006)	0	2	2	2	2	2	2	2	2	2	2	20	91%
Williams et al. (2005a)	1	2	2	2	2	2	2	2	2	2	2	21	95%
Williams et al. (2005b)	0	2	2	2	2	2	2	2	0	2	2	18	81%
Williams et al., (2014)	2	2	2	2	2	2	2	2	2	2	2	22	100%
Yerys et al., (2011)	2	2	2	2	2	2	2	2	2	2	2	22	100%

Note: 2 = Yes, 1 = Partial, 0 = No, N/A = Not applicable.

## Discussion

The analyses demonstrated relatively large and statistically robust overall effect sizes, indicating significantly impaired performance when investigating accuracy and error rate among individuals with ASD across age groups which is consistent with previous research [18, 58, 59].

Working memory deficits in ASD were found across diverse methods to measure WM and different outcomes of working memory. Therefore, the present study indicates that working memory deficits in ASD are independent of the specific modality of the task. The publication bias results suggest that studies of working memory in ASD are equally likely to be published regardless of magnitude or statistical significance. Therefore, the probability that these results would be altered by including unpublished studies, studies there were not in English; studies that did not consider IQ or compared ASD to another clinical population is low. These findings are consistent with our hypothesis that individuals with ASD experience impairments in WM and support the growing view that cognitive and executive abnormalities may be just as important as the core symptoms in ASD, which demonstrates the significance and the importance of investigating working memory in ASD and the difficulties arising from these deficits. Exploratory post hoc subgroup analyses were conducted to investigate the effects of sample characteristics on the effect sizes for each outcome. Moderator variables (age and IQ) however, did not explain a significant amount of the between study variation.

There are a number of differences between the current meta-analysis and the one conducted by Wang and colleagues. By adopting a more extensive search of the available literature and a more stringent inclusion criteria the results of the literature search of the current meta-analysis found 8868 studies in the initial search compared to the 499 studies found by Wang et al. Moreover, a number of studies considered in the current meta-analysis, which met the inclusion criteria of Wang and colleagues, were not included in their meta-analysis. The studies that are present in their meta-analysis and not in the current one are due to those studies not meeting our inclusion criteria of matching participants on IQ and age. Therefore, the results presented here provide a less biased, and more comprehensive, synthesis of studies examining working memory in ASD.

Wang and colleagues found that there was a significant impairment in WM in individuals with ASD when investigating accuracy. The moderation results showed that visuospatial WM was more impaired than verbal WM and cognitive processing (maintenance vs. maintenance plus manipulation) did not explain the severity of the impairment. While they did conduct a meta-regression on IQ and age and found that they are not predictors of the impairment in WM, it is unreliable to draw such a conclusion while not controlling for IQ and age in the meta-analysis, as some of the studies included in their analysis did not control for IQ and age between their participants (Table 7). Similar to Wang, we found difference in WM accuracy that were not moderated by IQ and age. However, we found a larger effect size in both visuospatial (*d*: -0.73) and phonological (*d*:-0.67) WM showing a medium effect size compared to Wang (visuospatial, *d*: −0.72 and phonological, *d*: −0.44) showing a medium and a low effect size. Since Wang converted error rate scores into accuracy, this is the first study to show differences in WM error rates. This shows that individuals with ASD make more errors on WM task compared to the TD controls. This is an important as a few studies show that while testing WM performances, ASD participants did not differ on their accuracy from the TD controls but when made more errors, demonstrating that accuracy is not the only way to identity WM weaknesses, which could mean that ASD individuals are not only have impairments choosing the correct response by identifying them as well.

While there was an observed effect size difference between visuospatial (*d*: -0.73) and phonological (*d*:-0.67), we could not run a subgroup meta-analysis as the data in the groups was not independent (i.e. the same study participants contribute to more than one of the subgroups in the forest plot; [55]). Wang and colleagues conducted meta-analysis on WM type (spatial vs verbal) although the data was also independent as evident from their forest plots, which is another concern with the validity of their findings. There may be multiple explanations for the suggested larger impairment in visuospatial memory compared to phonological memory impairment. It may be that visuospatial tasks are more challenging simply due to the task being less familiar for automatic response. Letters or numbers are typically used to test phonological memory and that may be one of the reasons that visuospatial memory exhibits more impairments, since phonological tests can be associated to spoken and written material that may be used or observed in everyday life.

Another explanation for the observed larger impairment in the visuospatial domain in ASD individuals is that there may be another underlying cause such as using different brain regions during WM tasks. Functional magnetic resonance imaging (fMRI) studies have demonstrated prefrontal cortex (PFC) activity during WM task performance [60, 61, 62, 63] and the left dorsolateral prefrontal cortex (DLPFC), a specific region of the PFC, is considered to play a crucial role in WM ([64, 65, 66], for meta-analytic reviews, see [67, 68, 69]). fMRI studies have also investigated WM in individuals with ASD, for example, Koshino et al.[70] examined brain activation of a group of adults with high-functioning autism during an n-back working memory task with letter. Their results demonstrated that individuals with ASD exhibited similar activation in the right hemisphere compared with the control group in contrast to substantially less activation in the left hemisphere in the dorsolateral prefrontal cortex and the inferior frontal gyrus. Individuals with autism showed more right lateralized activation in the prefrontal and parietal regions, whereas the control group demonstrated more activation in the left than the right parietal regions. In addition, individuals with ASD had more activation than the control group in the posterior regions including inferior temporal and occipital regions. Luna and college [71] investigated the abnormalities in prefrontal circuitry and their effects on spatial working memory, they found that individuals with ASD demonstrated significantly less task-related activation in dorsolateral prefrontal cortex and posterior cingulate cortex in comparison with healthy subjects during a spatial WM task. This has been supported further by multiple studies such as the studies by Vogan et al.[72] that investigated neural correlates of verbal WM using a one-back letter matching task with four levels of difficulty. They found that neural patterns of activations differed significantly between TD and ASD groups. TD group had activation in the lateral and medial frontal, as well as superior parietal brain regions, while the ASD group showed little recruitment of frontal and parietal regions. In addition, the study by Silk et al.[73] demonstrating that individuals with ASD displayed less activation in lateral and medial premotor cortex, dorsolateral prefrontal cortex, anterior cingulate gyrus, and caudate nucleus during a visuospatial mental rotation task. Future research should consider these observed differences in WM impairments and investigate them fully in order to clarify this issue.

Given that WM allows individuals to maintain information actively in a readily accessible format, various researchers have investigated its relationship with wider intellectual ability measures, such as fluid intelligence and scholastic aptitude [74]. Such research provides various viewpoints explaining the relationship between the two constructs. For instance, Engle et al. [24] and Colom, Flores-Mendoza, and Rebollo [75] investigated the WM- intelligence association and found that WM is strongly related with intelligence. In light of the WM correlation with intelligence, we ensured that our inclusion criteria included only studies that matched groups on IQ or there was no significant difference between the groups, thus, eliminating intellectual weakness as a cause of impaired WM. Therefore, the results of the study suggest that working memory deficit is not simply attributable to IQ deficits. However, Poirier et al., [76] note that when participant groups are matched on verbal IQ as measured by the Wechsler scales, group differences on WM tasks may be underestimated because the test on which participants are matched (i.e., the WAIS), includes a sub-test of short-term/working memory (the digit span). In other words, participants might partly be matched on the domain that is of interest. While Poirier and colleagues took this into consideration and matched their participants on WAIS scores that purposefully excluded the digit span sub-tests while other studies did not, thus, this could be a critical methodological issue that future studies should take into consideration.

### Strengths and limitations

Despite that there have been a number of comprehensive reviews of WM and ASD [18, 41, 58, 59], this is the first comprehensive review and meta-analysis of the current literature that investigates WM in ASD, while controlling for confounders such as age and IQ. We also divided WM into constructs, phonological and visuospatial, which consistent with the criterion recommendation [53], which is aimed to minimise heterogeneity and improve reliability in the results found. By conducting separate meta-analysis on the different possible outcomes of WM (accuracy and error rate) it allowed us to have a clear conclusion on the results of whether there are significant impairments in individuals with ASD. As the WM tasks used in experiments are not often identical, the search strategy used was vital. Using a large number of relevant key terms in the literature search allowed us to gain access to a wide range of studies. We ensured that our search strategy was inclusive of any studies that specifically state the testing of working memory despite the terminology used for the task. We also used the most commonly used understanding of working memory [17].

Using stringent criteria for inclusion in the meta-analysis lead the study to have some limitations. Some of the limitations of the study were that only published and English language studies were included in this review excluding studies that can potentially meet the inclusion criteria. Another limitation of the study was that we reviewed studies that tested older ASD individuals even though research has shown that WM is among the cognitive functions that decline with age [77, 78]. However, we felt it was important to investigate if the WM impairment is displayed across the life span of ASD individuals.

Due to the small number of studies included in this review, in particular phonological WM in ASD in comparison to matched TD, results should be interpreted with caution [55]. Furthermore, there are some factors that contributed to the large heterogeneity found in this meta-analysis. A large range of methods used to measure WM, and the outcome measured of each method (apart from accuracy and error rate). For example the study by Williams et al. [79] where they reported the accuracy mean as 570 for the ASD group using an N-back task compared to the study by Cui et al. [38] reporting the ASD group mean as 8 using a Block recall task. Another factor that could have contributed to the large heterogeneity is the is the different age groups used, for example the study by Gonzalez-Gadea et al. [80] looked at adults with a mean age of 33 in the ASD group and a mean of 38 in the control group, compared to the study by Ham et al. [81] where they looked at children with a mean age of 12 for the ASD and control groups. However, variation between studies is expected and was accounted for using the random effects model, which assumes heterogeneity.

It is important to note that the WM tasks across the studies were not matched and this must be considered when making any conclusion drawn from the current review as the results on the task may be influenced by psychometric properties of the test itself. In addition, individuals with ASD often have many comorbidities, such as Attention deficit hyperactivity disorder [82], learning difficulties (e.g. dyslexia) [83], and Obsessive-compulsive disorder [84]. Most of the studies in this review did not control for such confounders (or reported that they did) and thus these concurrent disorders may have contributed to the WM impairment observed.

### Theoretical and clinical implications

Nevertheless, despite the study's limitations, as evident by the effect size the findings from this study will have important implications for people with ASD. WM impairments impact upon academic achievement [85, 86, 87] because many academic activities depend on WM such as remembering instructions, solving problems (mental arithmetic), controlling impulses and focusing attention [88, 89]. Therefore, academic progress of children and young people with ASD may be impaired due to WM impairments described in this study. WM impairments also has an impact on everyday life as it is plays a crucial role for several everyday functions such as the development of theory of mind [90], navigation [91], every day problem solving [92], reading skills [93, 94] and language development [94]. Moreover, it has been demonstrated that WM deficiencies contribute to social problems in people with ASD [29] as it is necessary to keep social information constantly changing in WM for social flexibility [95]. WM also encodes emotions observed on faces [96], regulate emotional responses [97], slow learning [98] and learning disabilities [25], language development [19], and break from restrictive or repetitive behaviours [31]. Thus, clinicians should acknowledge that WM is significantly impaired and possibly a core issue in individuals with ASD. Additionally, clinicians should take into consideration that complains in regards to difficulties in everyday life from ASD patients could be related to the impairment of WM. The treatment of WM deficits could therefore improve some of the core cognitive and behavioural deficits characterising ASD.

### Future research

The findings of this study help extend the literature on ASD and can be used to develop future studies centred on the most effective way to improve memory and consequently enhance the quality of life for individuals with ASD. Future research should investigate the nature of severity of WM deficiencies in individuals with ASD while controlling for confounding factors, such as comorbid psychiatric or developmental disorders. In the future, it may be possible to examine the results from studies that include individuals with LFA to investigate whether the deficit is present across the spectrum, studies should also consider using larger sample size as many of the studies have a small sample size that could lead the study to being underpowered. Future studies can also investigate if parents or siblings of individuals with ASD also experience WM impairments.

### Conclusion

This review revealed that individuals with ASD display significant impairment in WM in both phonological and visuospatial domains across age groups this is important for the ASD population to help understand the disorder further and inform the development of interventions and intervention studies to improve WM in people with ASD.

## Supporting information

S1 Appendix Full search strategy of Medline database. (DOCX)

S1 Table Excluded studies. (DOCX)

S1 Checklist Prisma checklist. (DOC)

S1 References References of studies included in the meta-analyses. (DOCX)
