# Supplementary material for: A meta-analysis of working memory in individuals with autism spectrum disorders
Source: PLoS One. 2019 Apr 30;14(4):e0216198. doi: 10.1371/journal.pone.0216198 (PMC6490940; doi:10.1371/journal.pone.0216198)
Supplement: S1 Appendix — (DOCX) [file pone.0216198.s001.docx]

**S1 Appendix.** Full search strategy of Medline database

| **Medline** | | |
| --- | --- | --- |
| **No.** | **Search terms** | **Records** |
| 1 | autis*.tw. | 24594 |
| 2 | asperg*.tw. | 38742 |
| 3 | pervasive development* disorder*.tw. | 1694 |
| 4 | kanner*.tw. | 174 |
| 5 | childhood schizophrenia.tw. | 251 |
| 6 | exp child development disorders, pervasive/ | 23512 |
| 7 | Developmental Disabilities/ | 16182 |
| 8 | (PDD or PDDs or HFA or ASD or ASDs).tw. | 13093 |
| 9 | Rett*.tw. | 3165 |
| 10 | (language adj3 delay*).tw. | 1589 |
| 11 | (communicat* adj3 disorder*).tw. | 1795 |
| 12 | (speech adj3 disorder*).tw. | 2758 |
| 13 | 1 and 12 | 94672 |
| 14 | working memory.tw. | 18230 |
| 15 | WM.tw. | 6167 |
| 16 | working memory capacity.tw. | 881 |
| 17 | WMC.tw. | 248 |
| 18 | working memory span.tw. | 175 |
| 19 | short-term memory.tw. | 6215 |
| 20 | short-term memory span.tw. | 59 |
| 21 | reading span.tw. | 139 |
| 22 | listening span.tw. | 39 |
| 23 | digit span.tw. | 2017 |
| 24 | word span.tw. | 73 |
| 25 | letter span.tw. | 18 |
| 26 | spatial working memory.tw. | 2141 |
| 27 | Verbal working memory.tw. | 948 |
| 28 | Verbal WM.tw. | 154 |
| 29 | n-back working memory task.tw. | 106 |
| 30 | N-back.tw. | 873 |
| 31 | episodic memory.tw. | 4502 |
| 32 | 14 and 31 | 33625 |
| 33 | 13 and 32 | 492 |
