# Supplementary material for: A meta-analysis of working memory in individuals with autism spectrum disorders
Source: PLoS One. 2019 Apr 30;14(4):e0216198. doi: 10.1371/journal.pone.0216198 (PMC6490940; doi:10.1371/journal.pone.0216198)
Supplement: S1 Table — (DOCX) [file pone.0216198.s002.docx]

**S1 Table**. Excluded studies

| **Study** | **Reason for exclusion** |
| --- | --- |
| Gabig et al., 2008 | Participants were not match on IQ or there was a significant difference between the groups. |
| Geurts et al., 2004 | Participants were not match on IQ or there was a significant difference between the groups |
| Goldberg et al., 2005 | Participants were not match on IQ or there was a significant difference between the groups |
| Joseph et al., 2005 | Does not report participants full scale IQ. |
| Lopez et al., 2005 | Participants were not match on IQ or there was a significant difference between the groups |
| Luna et al., 2007 | Does not report means and standard deviation of groups |
| Nyden et al., 1999 | Did not measure IQ for the typical developed/control group. |
| Ozonoff et al., 2001 | Does not report means and standard deviation of groups |
| Russell et al., 1996 | Participants were not match on IQ or there was a significant difference between the groups. |
| Verte et al., 2005 | Participants were not match on IQ or there was a significant difference between the groups |
| Yi et al., 2014 | Has two typical develop/control groups, ability matched and age matched. |
| Zinke et al., 2010 | Participants were not match on IQ or there was a significant difference between the groups |
