# Supplementary material for: A meta-analysis of working memory in individuals with autism spectrum disorders
Source: PLoS One. 2019 Apr 30;14(4):e0216198. doi: 10.1371/journal.pone.0216198 (PMC6490940; doi:10.1371/journal.pone.0216198)
Supplement: S1 References — (DOCX) [file pone.0216198.s004.docx]

**S1 References of studies included in meta-analyses**

1. Brenner LA, Shih VH, Colich NL, Sugar CA, Bearden CE, Dapretto M. Time Reproduction Performance Is Associated With Age and Working Memory in High‐Functioning Youth With Autism Spectrum Disorder. Autism Research. 2015 Feb 1;8(1):29-37.
2. Crane L, Goddard L, Pring L. Autobiographical memory in adults with autism spectrum disorder: The role of depressed mood, rumination, working memory and theory of mind. Autism. 2013 Mar;17(2):205-19.
3. Cui J, Gao D, Chen Y, Zou X, Wang Y. Working memory in early-school-age children with Asperger’s syndrome. Journal of Autism and Developmental Disorders. 2010 Aug 1;40(8):958-67.
4. de Vries M, Geurts HM. Beyond individual differences: are working memory and inhibition informative specifiers within ASD?. Journal of neural transmission. 2014 Sep 1;121(9):1183-98.
5. García-Villamisar D, Sala SD. Dual-task performance in adults with autism. Cognitive Neuropsychiatry. 2002 Feb 1;7(1):63-74.
6. Geurts HM, Vissers ME. Elderly with autism: Executive functions and memory. Journal of Autism and Developmental Disorders. 2012 May 1;42(5):665-75.
7. Gonzalez-Gadea ML, Baez S, Torralva T, Castellanos FX, Rattazzi A, Bein V, Rogg K, Manes F, Ibanez A. Cognitive variability in adults with ADHD and AS: disentangling the roles of executive functions and social cognition. Research in developmental disabilities. 2013 Feb 1;34(2):817-30.
8. Ham HS, Bartolo A, Corley M, Rajendran G, Szabo A, Swanson S. Exploring the relationship between gestural recognition and imitation: Evidence of dyspraxia in autism spectrum disorders. Journal of Autism and Developmental Disorders. 2011 Jan 1;41(1):1-2.
9. Happé F, Booth R, Charlton R, Hughes C. Executive function deficits in autism spectrum disorders and attention-deficit/hyperactivity disorder: examining profiles across domains and ages. Brain and cognition. 2006 Jun 1;61(1):25-39.
10. Jiang YV, Capistrano CG, Palm BE. Spatial working memory in children with high-functioning autism: Intact configural processing but impaired capacity. Journal of abnormal psychology. 2014 Feb;123(1):248.
11. Joseph RM, Steele SD, Meyer E, Tager-Flusberg H. Self-ordered pointing in children with autism: failure to use verbal mediation in the service of working memory?. Neuropsychologia. 2005 Jan 1;43(10):1400-11
12. Kaufmann L, Zotter S, Pixner S, Starke M, Haberlandt E, Steinmayr-Gensluckner M, Egger K, Schocke M, Weiss EM, Marksteiner J. Brief report: CANTAB performance and brain structure in pediatric patients with Asperger syndrome. Journal of autism and developmental disorders. 2013 Jun 1;43(6):1483-90.
13. Koshino H, Kana RK, Keller TA, Cherkassky VL, Minshew NJ, Just MA. fMRI investigation of working memory for faces in autism: visual coding and underconnectivity with frontal areas. Cerebral cortex. 2007 May 20;18(2):289-300.
14. Landa RJ, Goldberg MC. Language, social, and executive functions in high functioning autism: A continuum of performance. Journal of autism and developmental disorders. 2005 Oct 1;35(5):557.
15. Maister L, Plaisted‐Grant KC. Time perception and its relationship to memory in autism spectrum conditions. Developmental Science. 2011 Nov 1;14(6):1311-22.
16. Minshew NJ, Goldstein G. The pattern of intact and impaired memory functions in autism. The Journal of Child Psychology and Psychiatry and Allied Disciplines. 2001 Nov;42(8):1095-101.
17. Nakahachi T, Iwase M, Takahashi H, Honaga E, Sekiyama R, Ukai S, et al. Discrepancy of performance among working memory‐related tasks in autism spectrum disorders was caused by task characteristics, apart from working memory, which could interfere with task execution. Psychiatry and clinical neurosciences. 2006 Jun 1;60(3):312-8.
18. Poirier M, Martin JS, Gaigg SB, Bowler DM. Short-term memory in autism spectrum disorder. Journal of abnormal psychology. 2011 Feb;120(1):247.
19. Sachse M, Schlitt S, Hainz D, Ciaramidaro A, Schirman S, Walter H, Poustka F, Bölte S, Freitag CM. Executive and visuo-motor function in adolescents and adults with autism spectrum disorder. Journal of Autism and Developmental Disorders. 2013 May 1;43(5):1222-35
20. Schuh JM, Eigsti IM. Working memory, language skills, and autism symptomatology. Behavioral Sciences. 2012 Nov 2;2(4):207-18.
21. Sinzig J, Morsch D, Bruning N, Schmidt MH, Lehmkuhl G. Inhibition, flexibility, working memory and planning in autism spectrum disorders with and without comorbid ADHD-symptoms. Child and adolescent psychiatry and mental health. 2008 Dec;2(1):4.
22. Solomon M, Ozonoff SJ, Ursu S, Ravizza S, Cummings N, Ly S, et al. The neural substrates of cognitive control deficits in autism spectrum disorders. Neuropsychologia. 2009 Oct 1;47(12):2515-26.
23. Steele SD, Minshew NJ, Luna B, Sweeney JA. Spatial working memory deficits in autism. Journal of autism and developmental disorders. 2007 Apr 1;37(4):605-12
24. Verté S, Geurts HM, Roeyers H, Oosterlaan J, Sergeant JA. The relationship of working memory, inhibition, and response variability in child psychopathology. Journal of Neuroscience Methods. 2006 Feb 15;151(1):5-14.
25. Williams DL, Goldstein G, Carpenter PA, Minshew NJ. Verbal and spatial working memory in autism. Journal of autism and developmental disorders. 2005 Dec 1;35(6):747.
26. Williams DL, Goldstein G, Minshew NJ. Impaired memory for faces and social scenes in autism: Clinical implications of memory dysfunction. Archives of clinical neuropsychology. 2005 Jan 1;20(1):1-5. b
27. Williams DL, Goldstein G, Minshew NJ. The profile of memory function in children with autism. Neuropsychology. 2006 Jan;20(1):21.
28. Williams DM, Jarrold C, Grainger C, Lind SE. Diminished time-based, but undiminished event-based, prospective memory among intellectually high-functioning adults with autism spectrum disorder: Relation to working memory ability. Neuropsychology. 2014 Jan;28(1):30.
29. Yerys BE, Wallace GL, Jankowski KF, Bollich A, Kenworthy L. Impaired Consonant Trigrams Test (CTT) performance relates to everyday working memory difficulties in children with autism spectrum disorders. Child Neuropsychology. 2011 Jul 1;17(4):391-9.
